# Strategic approaches to enhanced health service delivery for Aboriginal and Torres Strait Islander people with chronic illness: a qualitative study

**DOI:** 10.1186/1472-6963-12-143

**Published:** 2012-06-08

**Authors:** Clive Aspin, Ngiare Brown, Tanisha Jowsey, Laurann Yen, Stephen Leeder

**Affiliations:** 1 Poche Centre for Indigenous Health, University of Sydney, Sydney, Australia; 2 Menzies Centre for Health Policy, University of Sydney, Sydney, Australia; 3 Australian Primary Health Care Research Institute, Australian National University, Acton, Australia; 4 Menzies Centre for Health Policy, Australian National University, Acton, Australia

## Abstract

**Background:**

Aboriginal and Torres Strait Islander people with chronic illness confront multiple challenges that contribute to their poor health outcomes, and to the health disparities that exist in Australian society. This study aimed to identify barriers and facilitators to care and support for Aboriginal and Torres Strait Islander people with chronic illness.

**Methods:**

Face-to-face in-depth interviews were conducted with Aboriginal and Torres Strait Islander people with diabetes, chronic heart failure or chronic obstructive pulmonary disease (n-16) and family carers (n = 3). Interviews were transcribed verbatim and the transcripts were analysed using content analysis. Recurrent themes were identified and these were used to inform the key findings of the study.

**Results:**

Participants reported both negative and positive influences that affected their health and well-being. Among the negative influences, they identified poor access to culturally appropriate health services, dislocation from cultural support systems, exposure to racism, poor communication with health care professionals and economic hardship. As a counter to these, participants pointed to cultural and traditional knowledge as well as insights from their own experiences. Participants said that while they often felt overwhelmed and confused by the burden of chronic illness, they drew strength from being part of an Aboriginal community, having regular and ongoing access to primary health care, and being well-connected to a supportive family network. Within this context, elders played an important role in increasing people’s awareness of the impact of chronic illness on people and communities.

**Conclusions:**

Our study indicated that non-Indigenous health services struggled to meet the needs of Aboriginal and Torres Strait Islander people with chronic illness. To address their complex needs, health services could gain considerably by recognising that Aboriginal and Torres Strait Islander patients have a wealth of cultural knowledge at their disposal. Strategies to ensure that this knowledge is integrated into care and support programs for Aboriginal and Torres Strait Islander people with chronic illness should achieve major improvements.

## Background

Aboriginal and Torres Strait Islander people have worse health outcomes than other members of the Australian population, in common with the Indigenous populations of other developed countries such as New Zealand, Canada and the US [1-5]. These disparities are reflected in indicators that include lower life expectancy at birth, higher rates of death and illness, and higher rates of injury and disability among Aboriginal and Torres Strait Islander people than among other Australians [6,7]. Within this context of disadvantage, chronic illness is the major contributor to the current health disparities [6]. As an example, deaths from cardiovascular disease are three times higher for Indigenous Australians than they are for non-Indigenous Australians, with the disparity being most marked in younger age groups [8].

Across the country, diabetes is having a devastating impact on Indigenous communities with rates of diabetes three times higher among Indigenous people than among non-Indigenous people [9]. Indigenous Australians also have an earlier age of onset [6] and this places an additional financial burden on Indigenous Australians affected by this increasingly prevalent chronic condition [10,11].

Research shows that there is a link between poverty and obesity, a contributing factor to diabetes [12]. Moreover, diabetes increases one’s chances of having heart disease by as much as four to six times, while end stage renal disease, a common consequence of poorly managed diabetes, increases one’s risk of heart disease by twenty times [13]. With increasing rates of kidney disease in some Indigenous communities, we can expect to see this adding to the increasing burden of chronic illness among Aboriginal and Torres Strait Islander people [14].

Systemic problems related to racism and marginalisation have also had a negative impact on Indigenous health. Several studies have shown that Aboriginal and Torres Strait Islander patients continue to experience high rates of discrimination within the health sector [15,16] despite efforts to eliminate racism and improve access to health services [17].

These systemic problems are compounded by poor communication within the health system. The problems associated with poor communication [18,19] are exacerbated for Aboriginal and Torres Strait Islander patients, largely as a result of the inability of health care workers to understand and acknowledge the cultural background of Aboriginal and Torres Strait Islander people. Moreover, a history of poor access to health services as well as socio-economic disadvantage are likely to contribute to diminished levels of communication between health care workers and Aboriginal and Torres Strait Islander patients [20].

The current scarcity of Indigenous health workers adds a further layer of complexity to the challenges faced by Aboriginal and Torres Strait Islander people. Despite the evidence that health care workers from the same ethnic background as patients leads to improved health outcomes [21-24], little progress has been made in rectifying the current shortage of Indigenous health workers.

Indigenous patients have identified the absence of Indigenous workers as a significant barrier to the availability of health care [25]. While greater numbers of Indigenous people need to be recruited and retained, enhanced training programs in cultural awareness for non-Aboriginal health workers are also needed.

With rising rates of chronic illness and an ageing population, health systems will be confronted with mounting challenges and increasing demands on available services. These challenges may be even greater for communities confronted with barriers such as poor access to services[8]. This study provides insights from Aboriginal and Torres Strait Islander people affected by chronic illness and identifies strategies to confront the complexities associated with the growing epidemic of chronic illness within Indigenous communities in Australia.

## Methods

This study was conducted in partnership with two Aboriginal Medical Services (AMSs) that assisted with the recruitment of participants against our study criteria, obtaining their consent to be interviewed by a member of the research team. All participants were provided with written and oral explanations about the purpose of the research and their role in it. A partnership based on respect and equity was negotiated between the communities and the research workers [26], seeking to avoid the errors of non-partnered research with Indigenous peoples [27].

Ethical approval to conduct the study was granted by the Aboriginal Health and Medical Research Council of New South Wales, the Australian National University Human Research Ethics Committee, the ACT Health Human Research Ethics Committee, the University of Sydney Human Research Ethics Committee, and Sydney West Area Health Service Human Research Ethics Committee.

The data collection and analysis were carried out by a group of six researchers with backgrounds in health and social sciences who were involved in all stages of the research and also wrote this manuscript.

This paper reports key findings from the analysis of nineteen in-depth interviews with Aboriginal and Torres Strait Islander participants who had chronic illness and/or cared for a family member with chronic illness. Participants were recruited as part of the Serious and Continuing Illness Policy and Practice Study (SCIPPS), a large qualitative research project which investigated the experiences of people living with at least one of three index conditions, diabetes (n = 17), chronic obstructive pulmonary disease (n = 3) or chronic heart failure (n = 11).

Participants ranged in age from 34 to 70 and were recruited by purposeful sampling through referrals from AMSs and general practices in western Sydney, New South Wales, and the Australian Capital Territory and included people with one or more of the three index conditions. The wide age range was chosen because of the early onset of chronic illness and shorter life expectancy of Indigenous Australians [28].

Semi-structured in-depth interviews were conducted with participants, with interviews lasting from 45 to 90 minutes. Participants were asked to describe their experiences of living with chronic illness, and several questions focused on their experiences and interactions with health care services and workers.

We asked questions such as, "Can you tell me what it has been like for you living with diabetes/CHF/COPD?”, "How have your experiences with health professionals affected you?" and, "What could health professionals do that would most improve your care?" All participants also completed a brief demographic survey and provided information about their health conditions and health care encounters. The research team judged that sufficient data had been gathered when interviews were no longer providing new insights or ideas central to the experience of having DM/COPD/CHF, indicating data saturation.

All interviews were electronically recorded and transcribed *verbatim*. Transcripts and field notes were imported into QSR NVivo8 to assist data management and analysis [29]. The research team iteratively established a coding scheme based on analysis of the transcripts and this scheme was used to code all transcripts (each transcript was coded by four members of the research team to ensure rigour).

We used content analysis to identify matters commonly raised by participants [30] that related to the research questions. From the codes, broader themes or patterns were identified and these provided a framework for analysis. This step-by-step process of content analysis has been described by Braun and Clarke [31] and requires close and careful collaboration by members of the research team. Ongoing collaboration was viewed as an essential component of the study that began before the inception of the project and underpinned all stages of the project. By drawing on the multiple and diverse talents of the research team in a collaborative manner, this study adds to the qualitative evidence base related to Indigenous health.

Issues identified were explored thematically to understand the associations between particular topics [32]. In order to ensure robust analysis, members of the research team reviewed emerging themes throughout the analysis phase. Moreover, members of the research team were directly involved in the collection of data, analysis of the data and the reporting of findings and this meant that the review of themes was informed by insights gained during the various stages of the research process. Several themes or patterns were identified and these formed the key findings that we report here. They are closely linked to the question that this paper asks, “How do Aboriginal and Torres Strait Islander people experience chronic illness in relation to health services?”

This study has one important limitation. Because we recruited participants principally from AMSs, the experiences reported here may not apply to all Aboriginal and Torres Strait Islander people with chronic illness. This includes those people who access health services from non-Aboriginal and Torres Strait Islander services and especially those who do not have regular on-going relationships with primary health care services.

## Results

The participants in this study encountered a combination of negative and positive experiences as a result of their illness and struggled to achieve a balance in their lives as they sought to overcome the challenges posed by chronic illness. For some participants, the negative experiences far outweighed the positive and this was associated with highly critical views about the amount and quality of care and support that they received. For several, a history of prejudicial treatment was associated with negative views about health services and health care professionals. Overwhelmingly, however, participants reported factors which, when considered in combination, indicate a repository of cultural resilience that constitutes a form of protection against the harmful effects of sub-optimal care.

### Discrimination, stigma and prejudice

Many of the people we interviewed recounted stories from throughout their lifetime in which they experienced discriminatory treatment from agencies and people that included health care workers, educational services, banks, police, paramedics, welfare services, health and social services. Several drew on these negative experiences in their descriptions of the health services that they encountered as a result of having been diagnosed with a chronic illness. These were encapsulated by the observation made by a participant with diabetes,

"You do feel intimidated because you’ve copped it for so many years you’ve gone to hospitals, and they make you feel that little and make you feel like shit, that’s the way that I do feel and it’s left an imprint in my soul for years gone."

"(Participant B)^a^"

For this person, the history of negative treatment dates from early childhood. Moreover, for several participants, the impact of these types of experiences was intensified by the knowledge that family members, friends and peers also experienced similar prejudicial treatment.

When describing non-Indigenous health services, participants reported strong memories of experiencing discrimination, feeling patronised, and receiving inadequate access to services and poor quality of care.

"In the [non-Indigenous service] you're in, you're out. There's no friendliness … years ago they used to treat us differently through discrimination … they'd make us wait six or eight hours [for treatment] where the others would get in and out pretty quick … they judge you on your looks, your dress, and then how black you are."

"(Participant P)"

These kinds of experiences were strongly linked to feelings of frustration, sorrow, fear and distrust. An Aboriginal woman in her 50s described multiple experiences of this nature and the effect they had on her. In recounting a recent experience with a non-Indigenous eye specialist for the management of a diabetes-related condition, she said,

"I’ve had some really bad experiences with doctors here and I’ve told my [AMS] doctor about them. He [the eye specialist] was a total pig, you know, it’s just the way that they talk to you. The way they’re belittling you. Make you feel cheap and small and 'you’re wasting my time here'. You know? He was really, really rude and I mean he’s there to provide a service in a professional way and in a specialized area, he made me cry, no absolutely [I won't] go back to him."

"(Participant L)"

Experiences such as this led to some participants not absorbing the useful information supplied by these health professionals and resulted in failure to follow recommendations from health professionals. In this way, the message was not separated from the messenger. A man described this in the following way,

"Well I used to see [Specialist 1]. I don’t know if I should say but I just turned off. You know? But then [DR1] would say 'you want to go back and see him' because me sugar reading was bad, so I have to go and see him. But personally I didn’t – well see, I just switched off [because] he’s a smartie, I’ll go back to [DR1] you know? Cause I respect him. But [Specialist 1] I haven’t been to him for about 4 years, I think. Been a long time, when I was diagnosed 'you should do it regularly.' I know that but I don’t really want to go back."

"(Participant D)"

These negative experiences with specialists influenced participants' decisions not to follow up on doctor referrals, with these having negative implications for continuity and coordination of care.

Several participants framed this negative treatment in relation to the colour of their skin and made the claim that they were treated differently by health care workers (HCWs) because of it.

"It was our colour of our skins and the other woman that was with me, that was darker than me, that she judged us on the colour of our skin."

"(Participant S)"

Other participants reported incidents in which they considered that HCWs had made assumptions based on the colour of a person’s skin.

"They presumed because he was black and dribbling, because he’d had a massive stroke and couldn’t control his motions, that he was alcoholic."

"(Participant S)"

Not only did participants encounter such treatment by HCWs at all stages of their life cycle but they were also familiar with similar incidents in the lives of family members and friends. This combination of negative treatment within the health system had a profound impact on these people’s lives and, for many of them, meant that they avoided non-Indigenous health services and accessed all health care from Aboriginal services.

It is clear from this research that the views of Aboriginal and Torres Strait Islander people with chronic illness are overwhelmingly influenced by this assortment of negative treatment at the hands of non-Aboriginal HCWs and the services in which they work. As a consequence, patients either chose not to access services or established only a tenuous connection with those that they did access. Relationships such as these serve to compromise the quality of health care that Aboriginal and Torres Strait Islander people receive, with this resulting in poor health outcomes for people with chronic illness.

### Judgment and blaming

Participants' negative perceptions of health services were often exacerbated by the judgmental attitudes of staff they encountered in those services. Some participants recounted interactions with health care workers during which the worker blamed the patient for their health problem.

"It was offensive, her basic words was that why don’t you people, you’re supposed to be good about looking after your own, why don’t you take her back where she came from?"

"(Participant S)"

Judging people on appearances often led to misdiagnosis and inadequate quality of care. Several people in this study referred to incidents in which HCWs falsely assumed that an Aboriginal patient was intoxicated and failed to recognize symptoms associated with medical conditions such as stroke and diabetes.

"Even though he’d been flown there by helicopter, they presumed because he was black and dribbling, because he’s had a massive stroke and couldn’t controls his motions, that he was alcoholic, and they sent him there to dry out."

"(Participant S)"

Another participant made a similar comment, and offered advice about sound medical practice that should be applied to all patients rather than in a discriminatory manner.

"I think he’s just got this stereotype that even though I wasn’t drunk, I was disorientated and everything else. His assumption was I was drinking, he shouldn’t make assumptions of people. He should treat people for what the condition is there and then, not what he believes a condition is, without actually doing any investigation."

"(Participant O)"

### Poor communication

Several participants cited poor communication as a major obstacle to good care and support for Aboriginal and Torres Strait Islander people with chronic illness. Participants recalled times when patients were seen by HCWs who provided medical advice in terms that were difficult or impossible to understand. As an example, one participant observed,

"When they are being addressed, they’re not being spoken to in a way that they’re really understanding what the illness is, how they can control it, how they can contribute to looking after their own health."

"(Participant Q)"

As this person notes, poor communication can constitute a barrier to effective self-management of chronic illness. Moreover, this poor communication sometimes led to situations in which doctors made inappropriate diagnoses of medical conditions. A number of participants also cited examples of culturally insensitive practices and processes. Another participant noted that poor communication could lead to inappropriate delays in treatment.

"But when it comes to communicating about um, you know other things it’s, it’s, it’s always a, there’s always a wait. There’s all, you know and I’m talking 1 or 2 months. You know a dietician you’ve gotta wait 3 months to get in to."

"(Participant B)"

### Strategies to improve health outcomes

The people in this study described a number of strategies that could significantly enhance the standard of care and support for Aboriginal and Torres Strait Islander people with chronic illness. For most participants, these strategies need to be underpinned by a strong and visible Aboriginal and Torres Strait Islander workforce, recognition of the crucial role that family and peers play in the management of chronic illness and a desire by patients to be included and acknowledged in problem solving processes related to their chronic illness.

### Aboriginal and Torres Strait Islander health workforce

Recruiting greater numbers of Aboriginal and Torres Strait Islander people to the health workforce was seen by most participants as a high priority matter that would help to alleviate patients’ concerns and anxieties about their health care. The presence of Aboriginal and Torres Strait Islander staff in services such as the Aboriginal Medical Service (AMS) was cited as evidence of a service that was committed to improving Aboriginal and Torres Strait Islander health and well-being. Patients said that they felt accepted and that their health needs were taken seriously. Patients knew that they could approach such services with confidence and be assured that their health needs would be addressed in a culturally appropriate manner. Overwhelmingly, the participants in this study indicated strong support for the services that they received from the AMS. Most participants had long-term relationships with staff at the AMS and this was seen as a major factor in their decisions to seek health care from the AMS rather than other health services. Several cited particular HCWs as being instrumental in their decisions to access services at the AMS and to actively engage in health decision-making and self-management behaviour.

"I used to go to the medical centre in town, before this [AMS] was built, in that big old building over there, and I used to go all the way into [suburb], to see the AMS workers, and um I’d see a lot of people, it’s a great place to get together with a lot of new people, you know, a special place, and you see different ones that you know, and have a yarn to … I’ve been away for a while, and um I always come back, and the doctors are good. Everybody’s very good here."

"(Participant D)"

This positive appraisal of the AMS contrasted markedly with the negative interactions that many Aboriginal and Torres Strait Islander people experienced when they accessed non-Indigenous health services.

Participants also said that Aboriginal and Torres Strait Islander health workers played a key role in passing on information about the person’s chronic illness and this helped to minimize levels of anxiety and confusion that Aboriginal and Torres Strait Islander patients experienced as a result of being newly diagnosed with a chronic illness. As one person said, *“It was a whole new world.”* The information provided by health workers made an important contribution to the development of a patient’s health literacy and as such, contributed to improved self-management of the person’s chronic illness.

"She was like the social worker I guess, we could talk to them individually, she was lovely. She explained everything, she took you in to how you know it all worked and what was going to happen, and that you know, you couldn’t have found so much difference between her, and the doctors who just tell you, “Well, these are what’s going on with you, and if you don’t have dialysis, you’re going to be poisoned and that will kill you,” and that’s it, so clinical, I don’t know, I just felt shock."

"(Participant S)"

As this patient points out, the caring attitude of the health worker contrasted markedly with the clinical matter-of-fact attitude of the doctor and went a long way towards allaying the fears of the patient.

### Family, peers and social networks

Participants in this study attached great importance to the role that family members, peers and social networks played in the management of chronic illness.

The transmission of knowledge from one generation to another is an important aspect of Aboriginal and Torres Strait Islander culture and this featured prominently in the way in which participants perceived chronic illness and its impact on people and communities. Participants indicated that they played an important prevention role by passing on information about chronic illness to young people in the hope that this would help prevent them from being diagnosed with chronic illness as they grew older.

"And we were sitting there one night, and he says, “How can I get the message across to kids?” and I said “Well what about we have, you draw some animals and you get the animals to talk about the diabetes and how it affects you, how it makes you tired and just generally about diabetes?” So we actually did that and a 20 min DVD which we have given to lots of different medical centres."

"(Participant Q)"

At the same time, children often played an important role as advocates and support people for people with chronic illness. Several participants said that they relied on their children to intercede on their behalf during difficult encounters with health care workers. As an example, one person said,

"That was as far as I got because the daughter was standing on the other side of me, and she’s an entirely qualified person, and she basically said, “Don’t you dare accuse my mother. Dad’s been a diabetic for too long, and she’s fed him for too long for you to come in here and say something like that, so I think you’d better start cleaning up your act and check out, find out what it is”."

"(Participant M)"

Children also played an important role in helping patients to deal with everyday tasks such as shopping and preparing meals.

Several participants indicated that elders also played an important role in their day-to-day lives and the management of their chronic illness. In particular, the way that elders were treated within the health system had a profound impact on how participants viewed the health system and its ability to provide appropriate care and support. Some patients reported witnessing elders being treated disrespectfully in hospitals and palliative services. This contributed to the negative views that patients held about hospitals and health service providers.

"Anyhow, I went over and there was only six elderly people there from out, somewhere out there. Anyhow, they said they had no money or anything, so I …, but I just went and got them some bread and things like that. When I got back, they were very upset, they were sitting outside. And I said to them, “What’s wrong?” One of the sisters had gone in, and you know where the cancer ward is? [Staff] had removed the tea, coffee and milk, so [the elders] couldn’t have a cuppa."

"(Participant Q)"

Participants indicated that elders were instrumental in the dissemination of knowledge and information about health and well-being. As one person said, attendance at regular meetings of elders provided an important support role in the management of chronic illness.

"I go to these, we have a meeting, like the elders, every Friday, at the end of the month, I go to those. Yeah, the elders, like our age, and they, and ah the organs is diced up, like with sugar diabetes. I enjoy it, ‘cause it’s all gather round, you know, natter, have a good laugh and what, whatever. And yeah, it’s alright."

"(Participant D)"

### Long-term relationship with primary health care provider

Several participants cited a long-term relationship with one particular health care provider as being beneficial in the management of their chronic illness. In this study, the local AMS was identified as a health service that provided a range of medical and social services that had a positive impact on the health of participants. These participants said that they sought regular and ongoing care and support for their chronic conditions from their local AMS.

"It’s a great place to get together with a lot of new people, you know, a special placed and you see different ones that you know and have a yarn to, but I’ve been here ever since I’ve been ill, and I’ve been away for while and I always come back, and the doctors are good. Then everybody’s very good here."

"(Participant E)"

Other participants said that they liked visiting the AMS because it provided them with the opportunity to meet friends and other people which whom they could interact socially.

One participant said that he travelled a long distance to access an AMS. Despite the significant challenges that this posed, he preferred to attend an AMS rather than local mainstream health services. This illustrates the need for such services to be more easily accessible, especially to those Aboriginal and Torres Strait Islander people who live in rural locations.

Regular attendance at the same primary health care provider meant that patients were able to monitor their health closely and were able to receive referrals to specialist services in a timely and efficient manner.

## Discussion

This study is important because it is informed by the actual stories and experiences of Aboriginal and Torres Strait Islander people with chronic illness and their carers. From these accounts, it is clear that Aboriginal and Torres Strait Islander people with chronic illness confront a raft of barriers that contribute to poor health outcomes. These barriers relate to stigma and discrimination, and lead to inequitable access to treatment and an increase in risk factors for chronic illness.

In the face of these challenges, Aboriginal and Torres Strait Islander people with chronic illness are well equipped to play a central role in the management of their illness. With appropriate support mechanisms in place, and access to appropriate health services, Aboriginal and Torres Strait Islander people with chronic illness are able to manage their condition, with this leading to enhanced health outcomes.

### Enhanced Aboriginal and Torres Strait Islander health workforce

Aboriginal and Torres Strait Islander people are under-represented in the health workforce with this being mirrored among other Indigenous people in developed nations [6]. In New Zealand, for example, the proportion of Maori in the health workforce is less than that of non-Maori [33] Overwhelmingly, Indigenous people express high levels of satisfaction with health services provided by Indigenous health services and Indigenous health workers [34] with this being the case in our study. At the same time, they expressed considerable frustration about not being able to access Indigenous-specific services and Indigenous specialists when they needed to. As well, many Indigenous patients expressed displeasure about the type of service they received in non-Indigenous health services.

In order to meet the needs of Indigenous patients and improve health outcomes, it is imperative that significant efforts be made to improve the recruitment and retention of Indigenous people into the health workforce. Indigenous people with chronic illness would benefit from being able to access Indigenous health care workers with expertise in the management of chronic illness.

Despite many years of training in cultural awareness, health care professionals and health services still have much work to do to ensure that health care is delivered in a fair and equitable manner to all patients. This must be underpinned by significant investments in education and employment at all levels of the health system if there is to be any improvement in services for Aboriginal and Torres Strait Islander people with chronic illness in particular and for Indigenous communities in general. The effective recruitment and retention of Aboriginal and Torres Strait Islander workers into the health workforce must be the focus of efforts to improve Aboriginal and Torres Strait Islander health and contribute to a real reduction in disparities.

### Family networks

For many people with chronic illness, the management of their condition takes place within the context of family. Family members can play a variety of roles that can enhance the health of people with chronic illness. These roles include that of carer, companion, support person and advocate. Faced with a diverse array of specialist information about their illness and often complex medication regimens, patients rely heavily on the support of family members to assist in the effective management of their illness [35]. Family members need to be supported to provide effective care and support to patients with chronic illness since this is likely to provide benefits for the patients as well as relieve pressure and demand on health services [36].

Health services need to recognise the role that family members play in the lives of Aboriginal and Torres Strait Islander patients with chronic illness. This needs to be reflected in policies and procedures related to the care and support of people with chronic illness. As well, health care workers must be provided with appropriate training to ensure that Aboriginal and Torres Strait Islanders are supported in a culturally appropriate manner to develop effective self-management of their illness. Training must be designed and delivered in a way that ensures health care workers recognise and understand the role played by family members in the care and support of Indigenous people with chronic illness.

### Access to primary health care

Primary health care facilities with strong levels of community involvement can make a significant contribution to improved health outcomes for Indigenous people with chronic illness. An expansion of current primary health services with increased access for Indigenous patients would lead to significant health gains, with this contributing to enhanced prevention, and improved identification and management of chronic illness within Indigenous communities [37,38]. In a number of other countries with Indigenous populations such as New Zealand, Canada and the United States, there is evidence that primary health care has contributed to improved life expectancy of Indigenous peoples [39,40]. Moreover, the absence of primary health care is linked to an increase in the life expectancy gap between Indigenous and non-Indigenous people in the same country [41-44].

In response to these health imperatives, the Council of Australian Governments (COAG) committed to six specific goals, including that of “closing the gap”. These goals are supported by $4.6 billion of funding for initiatives in health, education, housing and employment. The report from the National Health and Hospitals Reform Commission has called for a streamlined funding system for Aboriginal and Torres Strait Islander health initiatives in order to strengthen the work of community-controlled organizations [45]. To ‘close the gap’ it is essential that the government act on this recommendation in order to ensure enhanced health outcomes through improved access to primary health care for Aboriginal and Torres Strait Islander people with chronic illness.

## Conclusion

These findings provide compelling evidence that current non-Indigenous health systems and health care services are failing to meet the needs of Aboriginal and Torres Strait Islander people with chronic illness. Moreover, these same health systems expose Aboriginal and Torres Strait Islander people to unacceptable levels of discrimination that undermine efforts to provide fair and equitable health care to all. As this paper has shown, Aboriginal and Torres Strait Islander people have a large repertoire of stories of negative treatment within the health system which have led to the development of an acute awareness of the failure of the health system to behave in a way that is fair and non-discriminatory.

As well as exposing inadequacies, Aboriginal and Torres Strait Islander people are also acutely aware of the ways in which health care provision can be improved. By drawing on knowledge and experience, the people in this study have identified strategic approaches that will enhance health services and contribute to improved health and well-being for Aboriginal and Torres Strait Islander people affected by chronic illness. Non-Indigenous health services in particular would benefit from drawing on these observations to improve the level of service that they provide to Aboriginal and Torres Strait Islander patients, families and communities. By focusing these initiatives on people affected by chronic illness, significant gains will be made and this will benefit generations to come. In the face of an increasing chronic illness epidemic, it is essential that these initiatives be implemented as a matter of urgency.

## Endnotes

^a^At the request of the health services with which we worked, we have been asked to use this term to refer to our informants in order to ensure anonymity.

## Competing interests

The authors declare that they have no competing interests.

## Authors’ contributions

CA led the drafting of the manuscript. TJ made a substantial contribution to the writing of the paper. NB provided cultural oversight to the development of the paper. CA, TJ, LY designed and implemented the study design, and participated in the recruitment, interviewing and analysis of data. All authors contributed to the writing and review of the manuscript and all authors have approved the final manuscript.

## Pre-publication history

The pre-publication history for this paper can be accessed here:

http://www.biomedcentral.com/1472-6963/12/143/prepub
